# Synthesis of Zinc Oxide Nanostructures on Graphene/Glass Substrate via Electrochemical Deposition: Effects of Potassium Chloride and Hexamethylenetetramine as Supporting Reagents

**DOI:** 10.1007/s40820-015-0045-5

**Published:** 2015-06-20

**Authors:** Nur Ashikyn Hambali, Abdul Manaf Hashim

**Affiliations:** Malaysia-Japan International Institute of Technology, Universiti Teknologi Malaysia, Jalan Sultan Yahya Petra, 54100 Kuala Lumpur, Malaysia

**Keywords:** Zinc oxide, Electrochemical deposition, Graphene, Nanorod, Nanowall

## Abstract

The effects of the supporting reagents hexamethylenetetramine (HMTA) and potassium chloride (KCl) mixed in zinc nitrate hexahydrate (Zn(NO_3_)_2_·6H_2_O) on the morphological, structural, and optical properties of the resulting ZnO nanostructures electrodeposited on graphene/glass substrates were investigated. The supporting reagent HMTA does not increase the density of nanorods, but it does remarkably improve the smoothness of the top edge surfaces and the hexagonal shape of the nanorods even at a low temperature of 75 °C. Hydroxyl (OH^−^) ions from the HMTA suppress the sidewall growth of non-polar planes and promote the growth of ZnO on the polar plane to produce vertically aligned nanorods along the *c* axis. By contrast, the highly electronegative chlorine (Cl^−^) ions from the supporting reagent KCl suppress the growth of ZnO on the polar plane and promote the growth on non-polar planes to produce vertical stacking nanowall structures. HMTA was found to be able to significantly improve the crystallinity of the grown ZnO structures, as indicated by the observation of much lower FWHM values and a higher intensity ratio of the emission in the UV region to the emission in the visible region. Equimolar mixtures of Zn(NO_3_)_2_·6H_2_O and the supporting reagents HMTA and KCl seem to provide the optimum ratio of concentrations for the growth of high-density, uniform ZnO nanostructures. The corresponding transmittances for such molar ranges are approximately 55–58 % (HMTA) and 63–70 % (KCl), which are acceptable for solar cell and optoelectronic devices.

## Introduction

The growth of highly crystalline metal oxides, such as zinc oxide (ZnO), on arbitrary substrates such as glass, metal, plastic and other conventional insulating materials, such as silicon dioxide and silicon nitride, is very difficult, primarily because of the large lattice mismatch [1, 2]. Because the structures of these arbitrary substrates and conventional insulating materials are amorphous or polycrystalline, ZnO structures that are grown on these materials also typically tend to be amorphous or polycrystalline. Graphene is a two-dimensional hexagonal network of carbon atoms that is formed through the establishment of strong triangular *σ* bonds of *sp* ^2^-hybridized orbitals [3, 4]. This bonding structure is similar to that of the *c* plane of a hexagonal crystalline structure and the (111) plane of the zincblende structure [5]. Therefore, the growth of ZnO on non-crystalline substrates using graphene as a template layer is feasible [6–9]. ZnO nanostructures and thin films on graphene are particularly interesting because these structures can imbue the graphene with additional functionality for the realization of advanced electronic and optoelectronic applications in photovoltaics, field emission devices, sensors, efficient energy conversion and storage devices, among others [10–14]. Graphene also offers considerable potential for novel electronic devices because of its extraordinary electrical, thermal, and mechanical properties, including a carrier mobility exceeding 10^4^ cm^2^ Vs^−1^ and a thermal conductivity of 10^3^ W mK^−1^ [15–19]. Therefore, by virtue of the excellent properties of graphene, the growth of ZnO nanostructures and thin films on graphene layers would enable the novel physical properties of both materials to be exploited in a wide variety of sophisticated device applications with flexible, wearable, and transferable capabilities [10, 20].

The most common methods of growing ZnO on graphene are vapor-phase techniques such as thermal evaporation [8, 9] and metal-organic vapor phase epitaxy (MOVPE) [21]. Vapor-phase methods are likely to involve high-temperature processes and are also considered to be high in cost. Additionally, because the growth process requires oxygen (O_2_), especially for the growth of ZnO via thermal evaporation, the possibility that the graphene will be oxidized or etched out during growth is high because graphene oxidation can occur at temperatures as low as 450 °C [22]. Liquid-phase methods, such as electrochemical or hydrothermal deposition, appear promising for the low-temperature growth of ZnO on graphene with well-controlled growth rates and structural dimensions [6, 7, 23, 24]. Although the growth of ZnO nanorods on graphene via a hydrothermal process has been reported, the need to use either seeded graphene or a pressurized container to induce growth is a significant disadvantage of this process. As an alternative, electrochemical deposition offers a feasible means of growing ZnO with high crystallinity and uniformity on a seedless graphene substrate with a simple process setup [6, 7, 24]. Recently, we have been examining the growth of ZnO nanorods on graphene on glass [25] because such a hybrid structure is expected to be promising for solar cell technology, which is a leading candidate for addressing the global need for inexpensive alternative energy sources.

Previously, we have reported the electrochemical growth of ZnO nanorods on graphene on glass using zinc nitrate hexahydrate as an electrolyte without any supporting reagents [25]. In that study, the effects of current density and temperature on the morphological, structural, and optical properties of the grown ZnO structures were investigated. The highest density of vertically aligned nanorods with few structural defects was obtained at a temperature and a current density of 75 °C and −0.1 mA cm^−2^, respectively. However, higher temperatures of above 80 °C appear to be required for the generation of nanorods with a well-defined hexagonal shape and a smooth top edge surface. In this work, the effects of supporting reagents, i.e., hexamethylenetetramine (HMTA) and potassium chloride (KCl), on the morphological, structural, and optical properties of the grown ZnO structures were systematically studied. The purpose of introducing these supporting reagents is not only to increase the conductivity of the solution used in the electrodeposition of the ZnO but also to enable the control of the resulting structures’ morphology. It is well known that because of the positions of the Zn and O ions in the ZnO unit cell and the asymmetry of the hexagonal lattice around the unit cell center, the wurtzite phase of ZnO exhibits a finite dipole moment along the hexagonal *c* axis. Because of this dipole moment, the ZnO (0001) surface becomes a “polar surface” and should be unstable. However, ZnO (0001) surfaces are commonly observed in ZnO nanostructures and thin films. Because of the presence of the dipole moment, the surface energy of the ZnO (0001) plane is higher than those of all low-index or non-polar planes, such as the (10$\bar{1}$0) and (2$\bar{1}\bar{1}$0) planes of the wurtzite ZnO crystal. The hydroxyl (OH^−^) and chlorine (Cl^−^) ions that are produced in the decomposition of HMTA and KCl, respectively, have different electronegativities; specifically, OH^−^ is less electronegative than Cl^−^. Therefore, their effects, particularly on the attraction/adsorption to the polar and non-polar planes of the ZnO crystal, should have a significant impact on the resulting morphological structures. Specifically, adsorption onto the side surfaces or non-polar surfaces of the ZnO will enhance the growth in the vertical direction, whereas capping on the basal plane or polar surfaces of the ZnO will promote the growth in the lateral direction.

## Experimental Procedures

A monolayer of graphene on glass (Graphene Laboratory Inc., USA) was used as a substrate for the growth of ZnO nanostructures. The electrochemical deposition process was performed via cathodic electrochemical deposition using two electrodes, with a platinum (Pt) wire acting as the anode and the monolayer graphene acting as the cathode [25]. A mixture of zinc nitrate hexahydrate (Zn(NO_3_)_2_·6H_2_O) (Sigma-Aldrich, ≥99.0 % purity) solution and a supporting reagent, i.e., either HMTA (C_6_H_12_N_4_) or KCl, was used as the electrolyte. Both the anode and cathode were connected to an external direct current (DC) power supply. In this experiment, electrodeposition was performed under galvanostatic control, with the current density remaining fixed during the deposition. The growth was performed at a current density of −0.1 mA cm^−2^ and a temperature of 75 °C because these conditions have been found to be optimal for obtaining the highest density of vertically aligned nanorods with few structural defects, as reported in Ref. [25]. The sample was inserted into the electrolyte at the beginning of the process, prior to the heating of the electrolyte from room temperature (RT) to 75 °C. Growth was allowed to continue for 45 min after the electrolyte temperature reached 75 °C. The grown structures were characterized using a field emission scanning electron microscopy (FESEM) apparatus (Hitachi SU8030) equipped with energy-dispersive X-ray (EDX) spectroscopy, X-ray diffraction (XRD, Bruker AXES D8 Advance), and photoluminescent (PL) spectroscopy (WiTec Alpha300R+) instruments and a UV–Vis spectrometer (Cary 5000).

## Results and Discussion

**Fig. 1:**
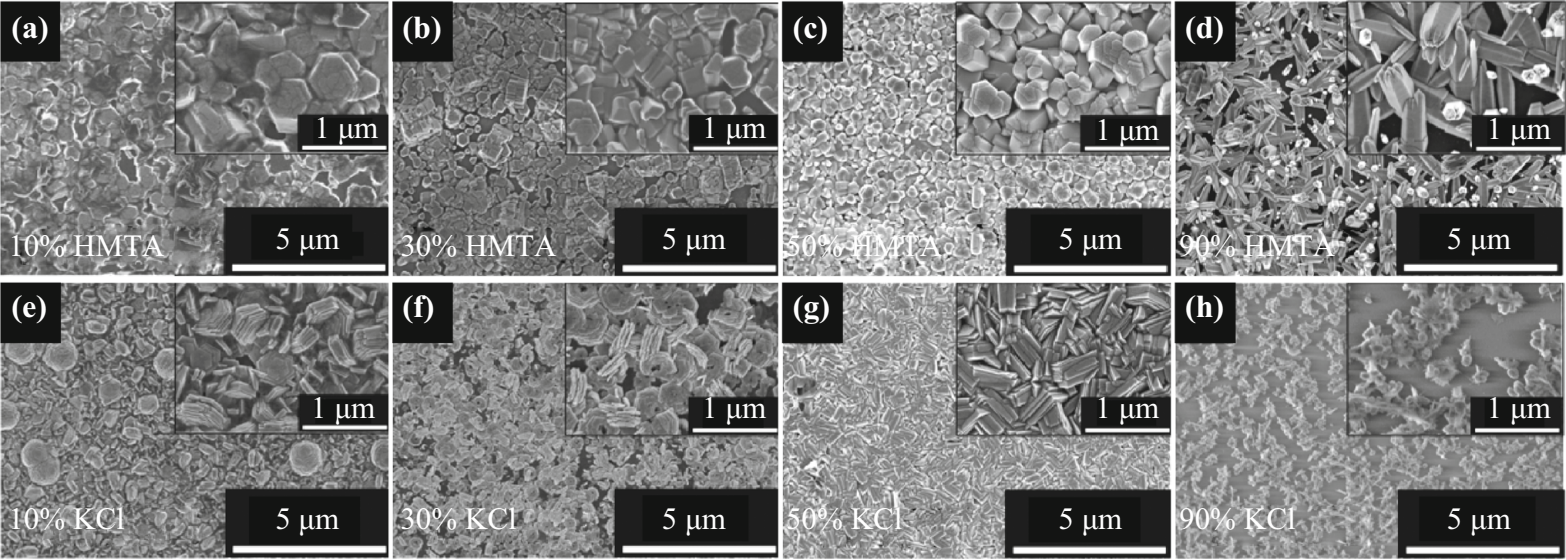
*Top-view* FESEM images of the ZnO structures grown in **a**–**d** HMTA-containing and **e**–**h** KCl-containing electrolytes

Figure 1a–d shows top-view FESEM images of the ZnO structures grown on graphene using mixtures containing different concentrations of HMTA, whereas Fig. 1e–f shows similar images of the nanostructures obtained using mixtures containing different concentrations of KCl. The molar ratio of Zn(NO_3_)_2_·6H_2_O to the reagent, i.e., HMTA or KCl, was set to 9:1 (denoted as 10 % HMTA or KCl), 7:3 (denoted as 30 % HMTA or KCl), 1:1 (denoted as 50 % HMTA or KCl), and 1:9 (denoted as 90 % HMTA or KCl). Figure 1 shows that in general, an electrolyte mixed with HMTA tends to produce nanorods that are hexagonal in shape, whereas an electrolyte mixed with KCl tends to produce stacking nanowall structures. The densities of nanorods and nanowalls also appear to increase as the reagent concentration is increased from 10 to 50 %, as shown in Fig. 1a–c and Fig. 1e–g, respectively. However, the densities of the grown structures decrease once again when the reagent concentration in the electrolyte is further increased to a high value of 90 %, as shown in Fig. 1d, h. Furthermore, at a reagent concentration of 90 %, in the case of HMTA, the grown nanorod structures exhibit poorly defined hexagonal shapes with rough top edge surfaces, as shown in Fig. 1d, whereas in the case of KCl, thin and sparsely distributed nanocluster-like structures are observed. The densities of the nanorods grown using electrolytes containing 10, 30, 50, and 90 % HMTA were estimated to be approximately 2.90 × 10^8^, 4.15 × 10^8^, 5.39 × 10^8^, and 2.28 × 10^8^ cm^−2^, respectively; these values are one order of magnitude lower than the nanorod density (1.45 × 10^9^ cm^−2^) obtained using an electrolyte without HMTA at the same current density and temperature [25]. The nanorod density was determined by averaging the quantities of nanorods observed in three different regions on each sample, with a total area of 125 μm^2^ for each region; the value thus obtained was then normalized to square centimeters (cm^2^). To determine the numbers of nanorods in such large regions, the numbers of nanorods observed in five FESEM surface morphological images were summed, where each image had dimensions of approximately 5 μm × 5 μm. In an analysis of the EDX spectra, only zinc (Zn), oxygen (O), and carbon (C) elements were detected in all of the grown samples, and the total compositional atomic percentages of Zn and O were estimated to be above 95 %.

The basic cathodic electrodeposition reactions of ZnO formation from a Zn(NO_3_)_2_·6H_2_O solution can be expressed as follows [6, 7, 25]:

Cathode:

1 $$ {\text{Zn}}\left( {{\text{NO}}_{ 3} } \right)_{ 2} \, \to {\text{ Zn}}^{ 2+ } +^{{}} 2 {\text{NO}}_{ 3}^{ - } $$

2 $$ {\text{NO}}_{ 3}^{ - } + {\text{ H}}_{ 2} {\text{O}} + 2\text{e}^{ - } \to {\text{ NO}}_{ 2}^{ - } + {\text{ 2OH}}^{ - } $$

3 $$ {\text{Zn}}^{ 2+ } + {\text{ 2OH}}^{ - } \to {\text{ Zn}}\left( {\text{OH}} \right)_{ 2} $$

4 $$ {\text{Zn}}\left( {\text{OH}} \right)_{ 2}\, \to {\text{ ZnO }} + {\text{ H}}_{ 2} {\text{O}} $$

Anode:

5 $$ {\text{H}}_{ 2} {\text{O }} \to \, \raise.5ex\hbox{$\scriptstyle 1$}\kern-.1em/ \kern-.15em\lower.25ex\hbox{$\scriptstyle 2$} {\text{ O}}_{ 2} + {\text{ 2H}}^{ + } + 2\text{e}^{ - }. $$

With reference to the above reactions, additional hydroxyl (OH^−^) ions are further obtained via the chemical reaction of HMTA with water, as shown below [6, 7].

6 $$ {\text{C}}_{6} {\text{H}}_{12} {\text{N}}_{ 4} + {\text{6H}}_{2}{\text{O}} \to {\text{COH}}_{ 2} + {\text{4NH}}_{3} $$

7 $$ {\text{NH}}_{3} + {\text{H}}_{2} {\text{O}} \to {\text{ NH}}_{4}^{+} + {\text{OH}}^{ - }. $$

The FESEM images shown in Fig. 1a–d suggest that HMTA percentages of approximately 50 % and below are most favorable for allowing the HMTA to act as a mineralizer to supply additional OH^−^ ions during the formation of the ZnO structures, thereby giving rise to nanorods with well-defined hexagonal shapes and smooth top edge surfaces [6, 7]. Based on the presented results, 50 % HMTA appears to be the optimal concentration, as it yields a high density of nanorods with good uniformity. Generally, it can be said that the adsorption of OH^−^ ions onto the side surfaces or non-polar surfaces of the ZnO enhances vertical ZnO growth, leading to nanorod formation. At low HMTA concentrations, it is speculated that the correspondingly small number of OH^−^ ions is not only insufficient to coordinate and bridge an adequate number of Zn^2+^ ions to form a uniform ZnO structure but also insufficient to attach to the non-polar side facets to facilitate vertical growth in the [0001] direction. This hypothesis is supported by the FESEM images shown in Fig. 1a, b, in which fewer vertical nanorod structures are observed. As the concentration of HMTA increases to 50 %, it seems to create a balanced number of OH^−^ and Zn^2+^ ions to perform the described activities, resulting in high-density, uniform nanorod structures, as shown in Fig. 1c. However, at high HMTA concentrations, the presence of excess OH^−^ ions compared with Zn^2+^ seems to create an unbalanced condition in which the excess OH^−^ ions play a significant role in coordinating and bridging Zn^2+^ ions to form ZnO structures rather than attaching to the non-polar side facets to facilitate the vertical growth in the [0001] direction, thus resulting in the formation of fewer vertically aligned nanorods, as shown in Fig. 1d.

When the KCl reagent was added to the electrolyte, instead of nanorods, vertically aligned nanowall structures were obtained. As mentioned in the previous section, ZnO has two types of crystal planes, which are classified as polar [i.e., (0001)] and non-polar [i.e., (10$\bar{1}$0) and (2$\bar{1}\bar{1}$0)] crystal planes. The highly electronegative chlorine (Cl^−^) ions generated in the chemical reactions with KCl should be easily attracted to the polar (0001) ZnO crystal plane. Once a layer of Cl^−^ ions has adsorbed and formed on the (0001) crystal plane, the subsequent deposition of ZnO can occur only on the non-polar crystal planes, which causes the individual ZnO hexagonal crystals to grow sideways, forming two-dimensional (2D) structures, i.e., stacking nanowall structures [26]. This expectation seems to indicate that Cl^−^ ions could act as a capping agent on the (0001) plane and thus control the formation of 2D nanowall ZnO structures, promoting growth toward the non-polar planes [26, 27]. It is speculated that at low KCl concentrations the correspondingly small number of Cl^−^ ions may be insufficient to attach to the polar side facets to facilitate lateral growth in the [10$\bar{1}$0] and [2$\bar{1}\bar{1}$0] directions. This hypothesis is supported by the FESEM images shown in Fig. 1e, f, in which fewer vertical nanowall structures and several vertical nanorods are observed. As the KCl concentration increases to 50 %, the grown structures become dominated by vertical nanowall structures with improved uniformity, as shown in Fig. 1g, suggesting that this is the optimal amount of Cl^−^ for achieving capping activity on the polar surfaces. However, at a high KCl concentration, the existence of an excess number of Cl^−^ ions compared with Zn^2+^ appears to create unbalanced conditions in which excess capping activity may inhibit ZnO nucleation, thereby resulting in the formation of thin and sparsely distributed ZnO nanoclusters, as shown in Fig. 1h. These results suggest that approximately 50 % of KCl is most favorable to act as a capping agent to suppress the growth of ZnO in the polar plane, resulting in the growth of ZnO toward the non-polar planes.

**Fig. 2:**
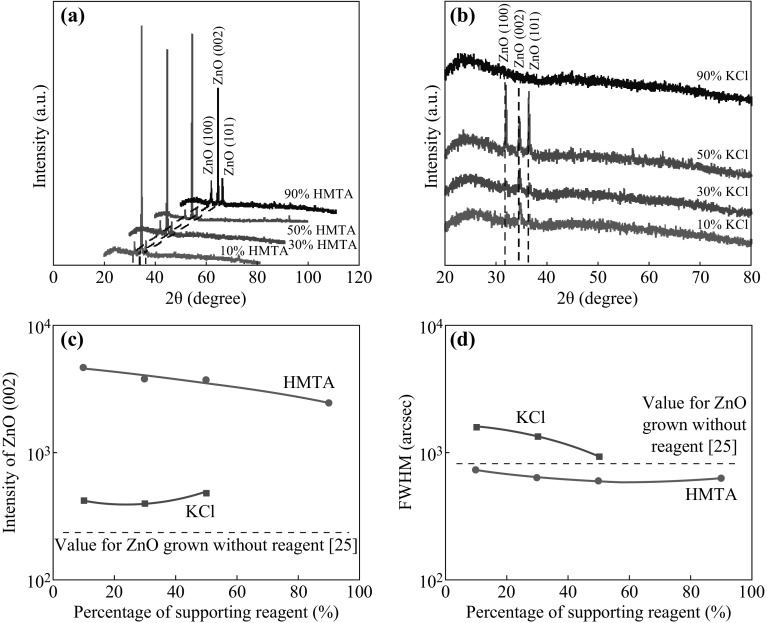
XRD patterns of the ZnO structures grown in **a** HMTA-containing and **b** KCl-containing electrolytes. **c** Intensities and **d** FWHM values of the ZnO (002) peak

Figure 2a, b shows the XRD spectra of the ZnO structures electrodeposited in the HMTA- and KCl-containing mixtures, respectively, with various concentrations. The XRD patterns show that the grown ZnO exhibits wurtzite structures, as indicated by the observation of the three main (100), (002), and (101) peaks of ZnO (JCPDS Card No. 36-1451). As shown in Fig. 2a, the intensity of the ZnO (002) peak is much higher than those of the other ZnO peaks, indicating the highly oriented growth of the ZnO along the *c* axis, particularly for the nanorod structures grown in the HMTA-containing mixtures. The formation of *c*-axis-oriented crystal structures can be attributed to the stabilization of the polar (0001) ZnO surface and the existence of additional OH^−^ ions suppressing the sidewall growth, resulting in the enhancement of ZnO formation along the direction of the polar surface. As shown in Fig. 2b, for the ZnO structures that formed in the presence of KCl, the intensity of the ZnO (002) peak is much weaker and the intensities of the other two peaks, i.e., (100) and (101), are relatively strong, especially for a KCl concentration of 50 %. This finding is attributed to the promotion of growth toward the non-polar direction, i.e., the formation of nanowall structures, as revealed by the FESEM images. However, no peaks can be detected for the samples grown at 90 % KCl, most likely because of the low density and low crystallinity of the thin and sparsely distributed nanocluster structures that were grown under these conditions. Figure 2c compares the intensities of the ZnO (002) peaks for the samples grown in HMTA- and KCl-containing mixtures with various concentrations. It is clearly observed that the intensities of the (002) peak for the samples grown using HMTA are much higher than those for the samples grown using KCl. It is also evident that for both HMTA and KCl individually, the intensities exhibit no significant difference among the tested concentrations. Moreover, the intensities of the ZnO (002) peaks of structures grown with supporting reagents are much higher than those of structures grown without any supporting reagent, as presented in Ref. [25], for the same current density and temperature.

Figure 2d compares the FWHM values of the (002) peaks for the corresponding grown structures. All samples grown in HMTA-containing mixtures exhibit lower values compared with the corresponding sample grown in a mixture without a reagent [25]; however, this finding simply indicates the better crystallinity of the structures grown using HMTA as a supporting reagent for the same current density and temperature. Here, the following important conclusions can be drawn: (i) the supporting reagent HMTA does not increase the density of the grown nanorods, but it does improve the smoothness of the top edge surfaces of the nanorods, even at a low temperature of 75 °C; (ii) HMTA suppresses the sidewall growth, i.e., the growth along the non-polar planes, while also promoting the growth of ZnO on the polar plane to produce vertically aligned nanorods along the *c* axis; (iii) by contrast, the supporting reagent KCl suppresses the growth of ZnO on the polar plane, i.e., along the *c* axis, while promoting the growth on the non-polar planes to produce vertically stacking nanowall structures; (iv) HMTA tends to increase the thickness of the grown structures due to the enhancement of the growth along the *c* axis, as indicated by the observation of a high-intensity (002) peak, whereas KCl cannot significantly increase the thickness because it enhances the growth toward the non-polar planes; and (v) HMTA is able to improve the crystallinity of the grown ZnO structures, as indicated by the observation of lower FWHM values.

**Fig. 3:**
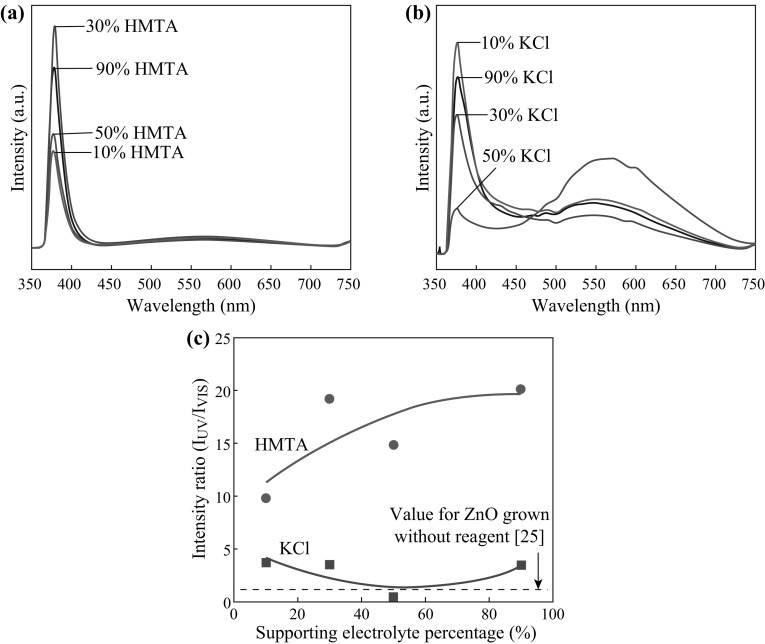
PL spectra of the ZnO structures grown in **a** HMTA-containing and **b** KCl-containing electrolytes. **c** Intensity ratio of the UV emission to the visible emission, *I*_UV_/*I*_VIS_

Figure 3a, b shows the RT PL spectra of the ZnO nanostructures grown on graphene with the addition of HMTA and KCl, respectively, as supporting reagents at various concentrations. Two distinct emission bands can be observed. The first band is located in the ultraviolet (UV) region, with peaks in the range of 376–378 nm, and the other is located in the visible region, with peaks in the range of 541–571 nm. This UV emission is also known as near-band edge (NBE) emission and can be regarded as an intrinsic property of the wurtzite crystal structure of ZnO, originating from exciton recombination [28]. The second emission band, in the visible region, has been reported to be related to the radial recombination of photon-generated holes with the single ionized charges of local defects such as O vacancies or Zn interstitials [29–32]. A stronger UV emission peak indicates good optical properties, which may be attributed to a lower defect concentration. Figure 3c summarizes the relative RT PL intensity ratio of the emission in the UV region to that in the visible region, denoted by *I*_UV_/*I*_VIS_. All structures grown with the addition of reagents exhibit much higher intensity ratios compared with the corresponding structures grown without the addition of a supporting reagent [25]; this is particularly pronounced for the samples grown using HMTA-containing mixtures. The samples grown with the addition of HMTA also exhibit higher values of the intensity ratio compared with the samples grown with the addition of KCl. Thus, it can be concluded that the use of HMTA-containing mixtures for ZnO growth tends to result in fewer structural defects in the obtained ZnO nanostructures [33, 34].

**Fig. 4:**
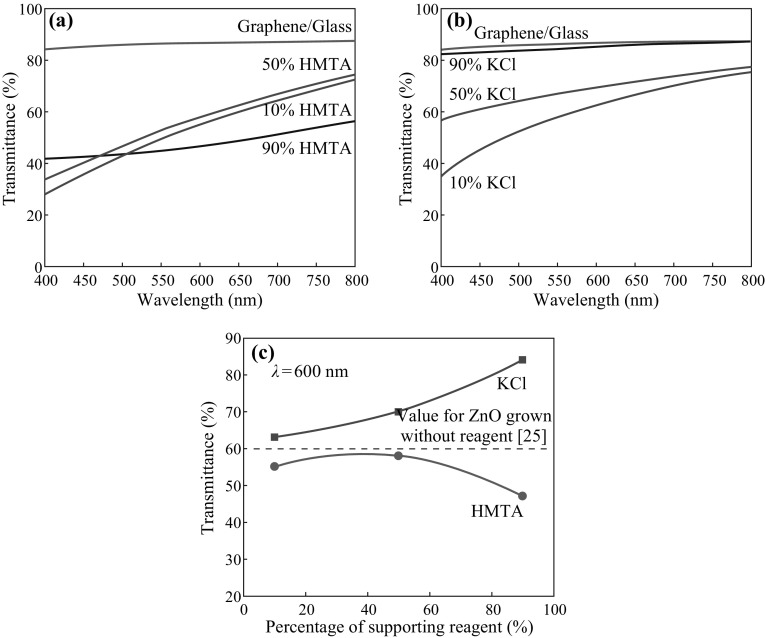
Optical transmittances of the ZnO structures grown in **a** HMTA-containing and **b** KCl-containing electrolytes. **c** Transmittance at a wavelength of 600 nm

Figure 4a, b shows the optical transmittance spectra of the ZnO structures grown in mixtures containing various concentrations of HMTA and KCl, respectively. The relationship between the supporting reagent concentration and the corresponding transmittance at a wavelength of 600 nm is shown in Fig. 4c. It was shown in the previous section that the optimal percentages of both reagents for the production of uniform ZnO structures are approximately 50 % and below. As shown in Fig. 4c, the corresponding transmittances for these molar ranges are approximately 55–58 % (HMTA) and 63–70 % (KCl), which are acceptable for solar cell and optoelectronic devices. The transmittances of the ZnO structures grown with the addition of HMTA are lower than those of the structures grown without the addition of a reagent [25]; this is most likely attributable to the greater thicknesses of these structures. The structures grown with the addition of KCl exhibit higher transmittance values compared with the other structures, i.e., those grown with the addition of HMTA or without the addition of a reagent. This difference can presumably be attributed to the fact that although the density of the nanowall structures is high, these structures are also thinner. No significant difference is observed between the samples grown at the lowest (10 %) and highest (90 %) HMTA concentrations, indicating that HMTA addition does not strongly affect the thickness of the obtained ZnO nanostructures. The high value of transmittance observed for the sample grown with 90 % KCl is most likely attributable to the low density and sparse distribution of the thin nanocluster structures.

Finally, it is worthwhile to briefly consider the other possible amines that can be explored for use as reagents to increase the density of vertically aligned nanorods. Polyethylenimine (PEI), a non-polar polymer with many amino side groups (–NH_2_), has the ability to become protonated, thereby becoming positively charged, over a wide range of pH values (3–11). Therefore, adjusting the pH of the growth solution to fall into the range that leads to the protonation of PEI can result in highly positive PEI that adsorbs strongly onto the negatively charged surfaces of the ZnO due to electrostatic attraction. It is well known that the isoelectric point of ZnO occurs at a pH of approximately 9.5; hence, the sign of the ZnO surface sites is predominantly positive or predominantly negative for pH values below or above the isoelectric point, respectively. Thus, it may be possible to enhance the vertical growth of high-density ZnO nanorods on graphene. Alternatively, citrate ions, which are characterized by three negative charges, are also good candidates for use as reagents to realize nanowall or nanoplate structures. Citrate ions may have the ability to strongly and specifically adsorb to the Zn^2+^ ions on the (0001) surface, thereby hindering the growth along the [0001] direction and forcing the growth to proceed along the [10$\bar{1}$0] and [2$\bar{1}\bar{1}$0] directions.

## Conclusions

The effects of the supporting reagents HMTA and KCl on the morphological, structural, and optical properties of ZnO structures grown on monolayer graphene substrates were systematically investigated. It was observed that the two reagents produce different morphological structures because of the different roles played by the OH^−^ ions from HMTA and the Cl^−^ ions from KCl. Equimolar concentrations of Zn(NO_3_)_2_·6H_2_O and the supporting reagents appear to be optimal for the growth of high-density ZnO nanorods and nanowalls. Moreover, adding a supporting reagent, especially HMTA, to the electrolyte for the growth of ZnO appears to improve the morphological and crystalline properties of the grown nanorods. The measured transmittances were approximately 55–58 % (HMTA) and 63–70 % (KCl), which are acceptable values for solar cell and optoelectronic devices. The obtained ZnO nanorods and nanowalls are also suitable to serve as low lattice mismatch seeds for the growth of gallium nitride (GaN) for the preparation of II–VI and III–V materials on insulators.
